# Learning challenges in children with brachial plexus birth injury: a preliminary study

**DOI:** 10.1093/pch/pxag054

**Published:** 2026-06-12

**Authors:** Kim Durlacher, Christine Hsin, Crystal Yu, Rebecca Courtemanche, Young Ji Tuen, Liisa Holsti

**Affiliations:** Department of Occupational Science and Occupational Therapy, University of British Columbia, Vancouver, British Columbia, Canada; Division of Plastic Surgery, British Columbia Children's Hospital, Vancouver, British Columbia, Canada; Department of Occupational Science and Occupational Therapy, University of British Columbia, Vancouver, British Columbia, Canada; Department of Occupational Science and Occupational Therapy, University of British Columbia, Vancouver, British Columbia, Canada; Division of Plastic Surgery, British Columbia Children's Hospital, Vancouver, British Columbia, Canada; Division of Plastic Surgery, University of British Columbia, Vancouver, British Columbia, Canada; Division of Plastic Surgery, British Columbia Children's Hospital, Vancouver, British Columbia, Canada; Division of Plastic Surgery, University of British Columbia, Vancouver, British Columbia, Canada; Department of Occupational Science and Occupational Therapy, University of British Columbia, Vancouver, British Columbia, Canada; British Columbia Children's Hospital Research Institute, British Columbia Children's Hospital, Vancouver, British Columbia, Canada

**Keywords:** Pediatrics, Brachial plexus, Birth injuries, Rehabilitation, Learning challenge, Learning disorder

## Abstract

**Objectives:**

To explore and describe learning challenges experienced by children with brachial plexus birth injury compared to the Canadian population.

**Methods:**

A retrospective chart review of 127 patients (mean age = 10 years 1 month, SD 5 years 1 month, *n* = 74 females) in a Canadian provincial brachial plexus clinic database was performed. Collected data included: patient demographics, birth history and complications, treatment interventions, and learning challenges. Data were summarized descriptively. The prevalence of learning challenges was compared with Canadian population data. Associations between reported learning challenges and patient and treatment characteristics were analyzed.

**Results:**

The per cent of learning challenges was 22.6% for males and 8.1% for females, compared to 4.0% and 2.5%, respectively, for the general population. Of those with a reported learning challenge, 56% had a formal diagnosis. Attention deficit hyperactivity disorder and speech delay were the most reported.

**Conclusion:**

This exploratory study found a higher prevalence of learning challenges in children with brachial plexus birth injury compared to Canadian children and youth. Further research is needed to corroborate and build on these initial results. Implementation of regular screening practices to support early detection of learning challenges may be considered to facilitate referral to specialty services with the goal of optimizing a child's health and well-being.

## Graphical Abstract

**Graphical Abstract:**
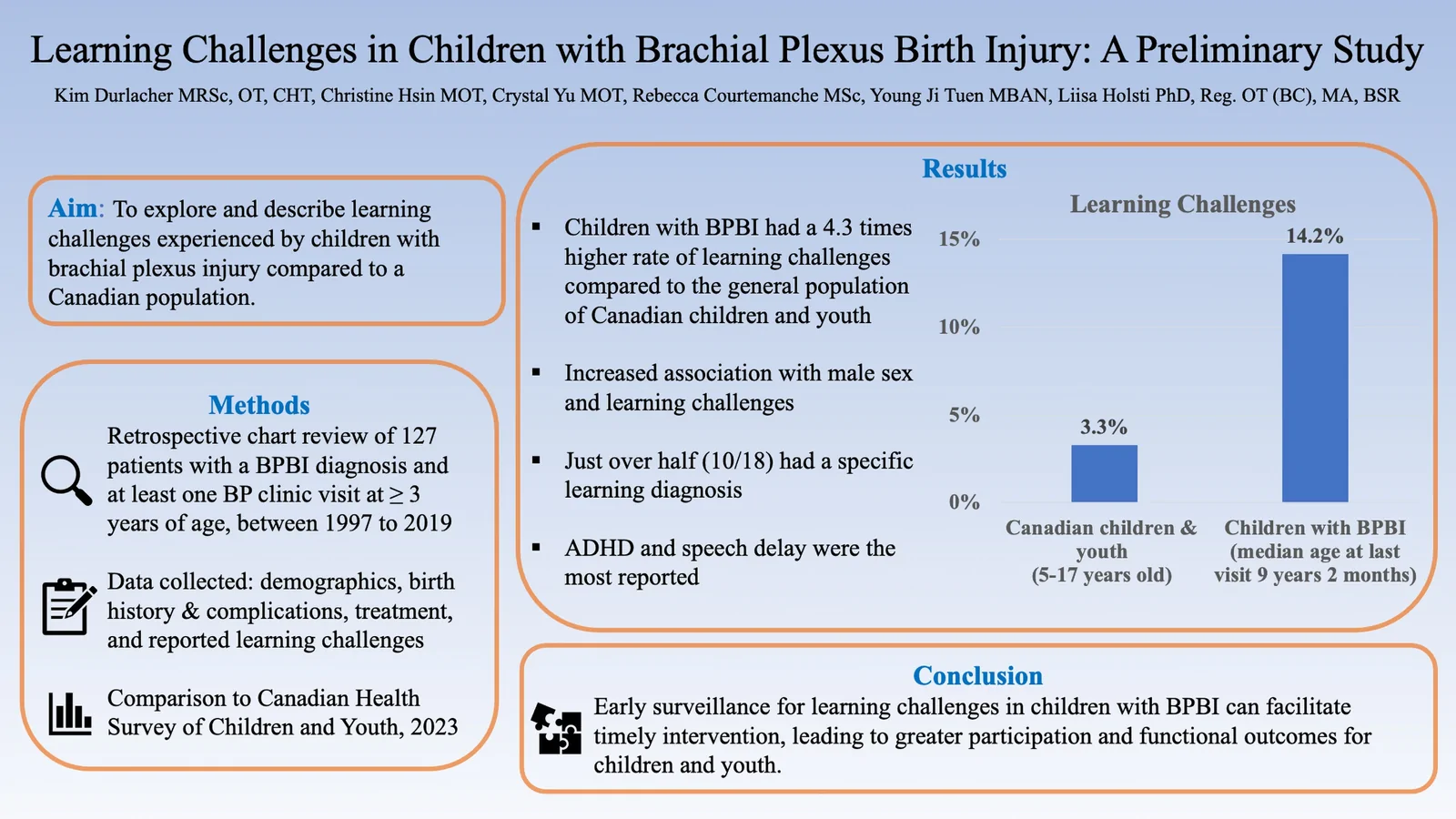

Brachial plexus birth injury (BPBI) involves damage to the brachial plexus (BP), a group of motor and sensory nerves that innervate the upper extremities (1). BPBI is one of the more frequent forms of birth trauma, with a reported incidence of 1.24 infants per 1000 live births in Canada (2). Severity can range from a stretch or compression injury to complete separation of the nerve(s) from the spinal cord; outcomes can range from spontaneous full recovery to lifelong impacts on a child's upper limb function (1, 3, 4). Where available, children may be followed over an extended period by a multidisciplinary BP clinic team.

Infants with BPBI may experience a range of perinatal and delivery risk factors including shoulder dystocia, macrosomia, the use of vacuum or forceps, prolonged labour, birth hypoxia, maternal gestational diabetes, and breech presentation (5–7). Some of these factors, such as birth hypoxia (8, 9) and maternal diabetes (10), are associated with neurodevelopmental concerns, including gross and fine motor differences, attention deficit hyperactivity disorder (ADHD), learning challenges, and autism spectrum disorder (ASD) (11). Other studies have reported a greater incidence of developmental and behavioural problems (3, 12) and language delays (13) in children with BPBI. Yet, there is a paucity of work exploring potential developmental or behavioural sequelae for these children (3, 12, 14).

Part of the complexity of this work may be, as Hayes et al (15) described, the struggle to define learning disabilities, with no official international recognition of the term, use of varying terminology, variations in severity and effect, and acknowledgement that not all learning challenges are caused by a defined disability. This has led to confusion with terms such as learning challenge, disability, or disorder used interchangeably, with differing intent amongst the medical, educational, and legal literature, which results in difficulties in comparing prevalence rates and resource needs for these children (16, 17). Additionally, some children may experience learning challenges without ever meeting the criteria for a formal diagnosis (18, 19).

Notably, the origins of a learning challenge are multifactorial and complex in nature and cannot be directly correlated to a BPBI itself. However, with awareness of potential risk factors and types and frequency of learning challenges experienced by BPBI patients, health care practitioners (HCPs) involved in the early years of a child's life may be well-positioned to facilitate timely screening. Early support and intervention for children with learning challenges is well documented for best outcomes in school/community participation and engagement (20, 21).

Given limited existing evidence, this preliminary study aimed to explore learning challenges in children with BPBI. Our specific objectives were to review all children with BPBI followed by a multidisciplinary team at a provincial tertiary paediatric center to (1) identify the percentage of children with any reported challenge affecting their learning, (2) describe the reported learning challenge(s), and (3) explore a comparison of the proportion of reported learning challenges in the BPBI study sample with the proportion of learning challenges in the Canadian population of children and youth.

## Methods

This study was a retrospective review of patients with a BPBI seen at the British Columbia Children's Hospital (BCCH) BP clinic, the only provincial multidisciplinary paediatric BP clinic. Ethics approval (H22-02054) was obtained from the University of British Columbia Children's and Women's Research Ethics Board. A waiver of consent was granted by the research ethics board.

### Participants

Eligible patients were identified from the clinic database. Inclusion criteria were a diagnosis of BPBI and at least one visit at ≥3 years of age between 1997 and 2019. This age-based criterion corresponds to an age when learning challenges may begin to appear (15). Exclusion criteria were a diagnosed genetic anomaly or near normal range of motion (ROM, thought to reflect a very mild injury requiring less follow-up over time) as measured by the active movement scale (AMS) (22). The criteria for near normal ROM were as follows:

- AMS scores ≥ 6 at or before 6 ± 2 months of age with no worsening of scores over time.
- First visit at ≥8 months of age with AMS scores ≥6 with no worsening of scores over time.
- AMS scores ≥ 6 on all movements except for supination, which was ≥4 at or before 6 ± 2 months of age, with no worsening of scores over time.

### Data collection

Patient demographics, birth history, delivery and complications, treatment of the BPBI, and age at last hospital visit were collected. Splint treatment for BPBI was defined as use of any positioning support of the affected arm including post-operative positioning.

Given the complexity of terminology around defining a learning disorder/disability and the exploratory nature of this study, we defined any documented symptom affecting the child's learning as a learning challenge. Hospital medical record (HMR) documentation pertaining to any learning challenge was collected, such as type, formal diagnoses, age noted, and family history of a learning challenge. Information on areas of challenge, such as emotional regulation, attention, written and verbal comprehension, speech, socialization, gross and fine motor skills, and everyday function, was recorded. During the study time period, there was no defined procedure to collect information on a child's learning challenge, thus only documented by the BP clinic if incidentally mentioned by caregivers.

Two authors (C.H., C.Y.) reviewed 10 charts together, and thereafter each reviewed 20 charts independently. To maintain consistency in data collection, this pattern was repeated for all charts. For every 50 charts, five were randomly selected for re-review to ensure quality control. Conflicts were resolved through discussion or consultation with other authors.

### Statistical analysis

Patients were grouped into those with reported learning challenges and those without. Patient demographics, birth complications, and treatments were classified descriptively using medians and interquartile ranges for numerical variables and counts for categorical variables. For children with reported learning challenges, their demographics, BPBI treatment, type or description of learning disability and/or challenge, as well as concurrent diagnoses, were summarized. Associations between a reported learning challenge and patient demographics, birth complications, or treatment characteristics were analyzed using Chi-square and Wilcoxon Rank Sum tests at a significance level of 0.05. Effect sizes were reported as phi for 2 × 2 chi-square tests and as rank-biserial correlation coefficients (*r*) for Wilcoxon rank-sum tests. Analyses were done using R Statistical Software Version 4.3.2 (23).

An exploratory comparison of learning challenges between the study sample and Canadian children and youth aged 5 to 17 years, as measured by the Canadian Health Survey of Children and Youth 2023, was conducted (24). In the survey, the person with the most knowledge about the child's learning abilities was asked, “Compared with children of the same age, does the [child/youth] have difficulty learning things?” (25) A two independent proportions analysis was performed, and results were reported with 95% confidence intervals.

## Results

A total of 394 patients were screened for eligibility, of which 267 patients were excluded (Figure 1). Of the 127 patients included, the median age was 9 years 2 months (IQR 6.0, 14.3), and 58% (*n* = 74) were female; information pertaining to family history of a learning challenge was limited by missing data (Table 1). Almost all patients had a unilateral injury (59% right-sided, 40% left-sided), and nearly half (*n* = 55, 43%) had one or more birth complications, of which the most common were respiratory (*n* = 48, 38%) or asphyxia (*n* = 18, 14%) (Table 2). All patients participated in physiotherapy, and 70% (*n* = 89) received splinting with or without surgery (Table 3).

**Figure 1:**
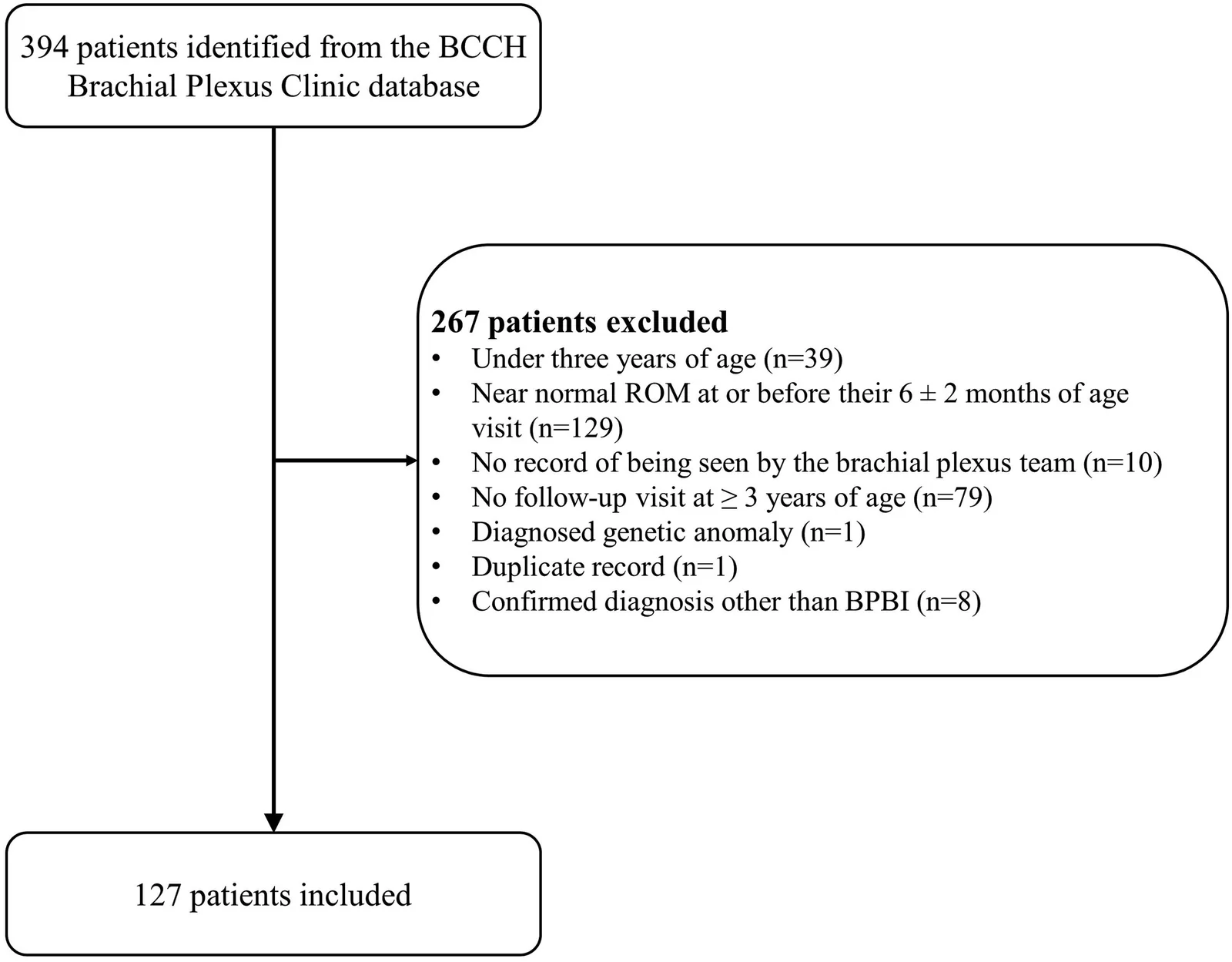
Study population.

**Table 1:** Patient characteristics and demographics.

	Children with learning challenges (*n* = 18)	Children without learning challenges (*n* = 109)	All (*n* = 127)
Age at last hospital visit (years)			
Median	10.63	8.58	9.17
[IQR]	[7.46, 16.15]	[5.92, 13.25]	[6.00, 14.29]
Sex at birth			
Male	12 (67%)	41 (38%)	53 (42%)
Female	6 (33%)	68 (62%)	74 (58%)
Side of injury			
Right	13 (72%)	62 (57%)	75 (59%)
Left	5 (28%)	45 (41%)	50 (40%)
Bilateral	0 (0%)	2 (2%)	2 (1%)
Family history of a learning challenge			
Yes	4 (22%)	2 (2%)	6 (5%)
No	3 (17%)	0 (0%)	
Missing	11 (61%)	107 (98%)	118 (93%)

IQR, interquartile range.

**Table 2:** Other birth complications of children with BPBI by documented learning challenge(s).

Other complication type	Children with learning challenges (*n* = 18)	Children without learning challenges (*n* = 109)	All (*n* = 127)
Asphyxia	2 (11%)	16 (15%)	18 (14%)
Respiratory	9 (50%)	39 (36%)	48 (38%)
Clavicle fracture	1 (6%)	3 (3%)	4 (3%)
Rib fracture	0 (0%)	0 (0%)	0 (0%)
Humerus fracture	0 (0%)	2 (2%)	2 (2%)
Horner's syndrome	0 (0%)	4 (4%)	4 (3%)
Shoulder subluxation	0 (0%)	1 (1%)	1 (1%)
Hemi-diaphragm paralysis	0 (0%)	1 (1%)	1 (1%)
Total complications	12	66	78
Patients with one or more other birth complications	11 (61%)	44 (40%)	55 (43%)

**Table 3:** Treatment beyond physiotherapy.

	Children with learning challenges (*n* = 18)	Children without learning challenges (*n* = 109)	All (*n* = 127)
BPEG	6 (33%)	29 (27%)	35 (28%)
Tendon transfer	6 (33%)	30 (28%)	36 (28%)
Distal nerve transfer	4 (22%)	13 (12%)	17 (13%)
Osteotomy	0 (0%)	10 (9%)	10 (8%)
Botox	4 (22%)	20 (18%)	24 (19%)
Splinting*	8 (44%)	43 (39%)	51 (40%)
Number of patients who received treatment beyond physiotherapy	13 (72%)	76 (70%)	89 (70%)

^*^Splinting may include Sup-ER splint (26), shoulder spica, wrist, hand, and/or elbow.

BPEG, brachial plexus exploration and grafting.

Eighteen (14.2%) patients had a documented learning challenge in their HMR noted at a median age of 5 years (IQR 3.5, 10). There were twelve males and six females with learning challenges, with a statistically significant association found for male sex (*p* = 0.040, *φ* = 0.183). A documented learning challenge was not associated with side of injury (*p* = 0.436, *φ* = 0.114), one or more additional birth complications (*p* = 0.189, *φ* = 0.117), treatment beyond physiotherapy (*p* = 1.000, *φ* = <0.001), or age at last hospital visit (*p* = 0.122, *r* = 0.230).

Of the 18 patients with documented learning challenges, ten had a formal diagnosis related to their learning. The most common was ADHD (*n* = 4) (Table 4). Patients with a learning challenge who did not have a formal diagnosis (*n* = 8) had a documented developmental delay in one or more areas (attention, gross motor, emotion, or speech/language) or were waiting for assessment of suspected ASD (Table 4).

**Table 4:** Characteristics of patients with reported learning challenges.

Patient ID	Sex at birth	Side of injury	Treatment beyond PT	Birth complications	Learning challenges documented	Formal diagnosis	Concurrent diagnoses
1	M	Left	No	Yes (respiratory)	Speech delay, challenges with dexterity and learning to read	ADHD	Myotonic dystrophy (diagnosed at age 16 years old)
2	F	Right	Yes	No	None noted	Written output disorder	Bipolar disorder
3	M	Right	No	Yes (asphyxia)	None noted	Learning disabilities, general	PTSD, mood disorder, ODD, tic disorder
4	M	Left	Yes	Yes (respiratory)	Difficulty staying still, fidgeting, blurting out answers in class, interrupting conversations, impulsive, difficulty with attention	Learning disability in auditory processing	
5*	M	Right	Yes	Yes (respiratory)	Behavioural difficulties	ADHD	
6	M	Right	No	Yes (asphyxia)	Difficulty writing, delayed development impacting communication, socialization, and language skills, speech delay	ASD	
7	F	Left	Yes	Yes (respiratory)	None noted	ADHD	Anxiety, depression
8*	M	Right	Yes	No	Concerns regarding social skills and behaviour, panic attacks	ASD	
9*	F	Right	Yes	No	Trouble focusing, required 1 to 1 support in preschool	ADHD	Tic disorder
10	M	Right	Yes	No	Impulsive, defiant, difficulties with attention	ADHD, Neurodevelopmental disorder	Separation anxiety disorder
11	M	Right	Yes	Yes (respiratory)	Hyperactivity, sensitivity to noise, enjoys solitary activities, restricted interests, echolalia	None noted	
12	F	Right	No	No	Difficulties with attention and defiance	None noted	Hypothyroidism, leukemia
13	M	Right	Yes	Yes (respiratory)	Developmental delay, gross motor	None noted	
14	M	Left	No	Yes (asphyxia)	Poor focus, speech, emotional regulation, developmental impairment	None noted	Visual impairment
15*	F	Right	Yes	No	Short attention span, academics well below grade level, expressive language delays, poor social skills	None noted	
16	M	Left	Yes	No	Speech delay (apraxia, expressive difficulties)	None noted	
17	F	Right	Yes	Yes (right clavicle fracture)	Anxiety, poor emotional regulation, separation, anxiety, waitlist for ASD assessment	None noted	Left congenital ptosis
18	M	Right	Yes	Yes (respiratory)	Waitlist for ASD assessment	None noted	

^*^Indicates family history of learning disabilities. ADHD, attention deficit hyperactivity disorder; ASD, autism spectrum disorder; ODD, oppositional defiant disorder; PTSD, post-traumatic stress disorder.

The prevalence of learning challenges in the study sample was 14.2%, which was 4.3 times higher (+10.9%, 95% CI 5.9%, 18.0%) than the prevalence of learning challenges in Canadian children and youth aged 5 to 17 years (3.3%) (24) (Figure 2). Males with BPBI in the study sample had a 5.7 times higher percentage of learning challenges compared to Canadian children and youth (22.6% vs. 4.0%, difference = 18.6%, 95% CI 9.5%, 31.5%), while females were found to have a 3.2 times higher percentage (8.1% vs. 2.5%, difference = 5.6%, 95% CI 1.3%, 14.1%).

**Figure 2:**
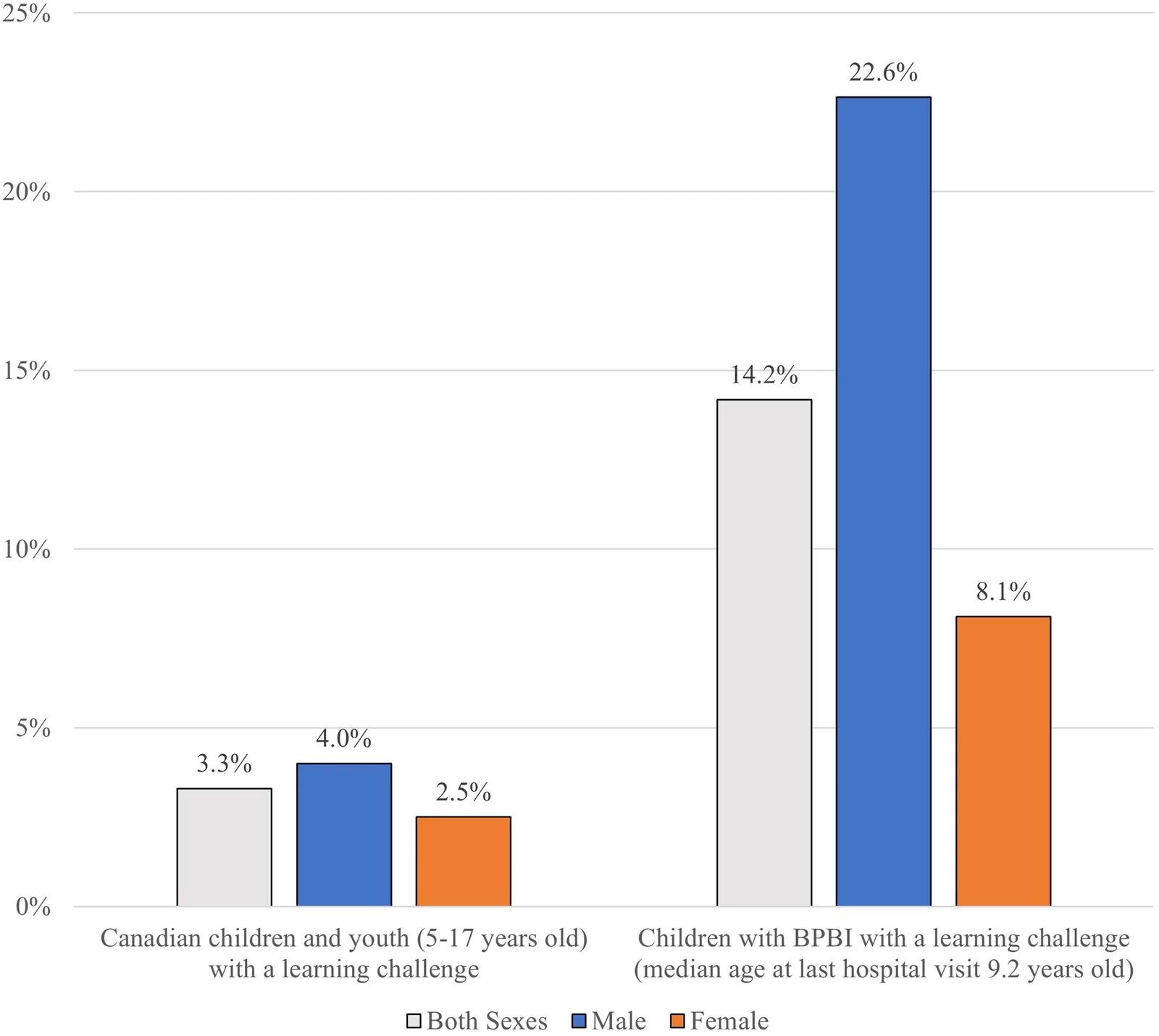
Canadian children and youth aged 5 to 17 years with a learning challenge (Canadian Health Survey on Children and Youth—2023 (25)) compared to children with BPBI with a learning challenge.

## Discussion

To our knowledge, this is the first study in Canada to explore learning challenges in children with BPBI. As previously noted, given the exploratory nature of this study and complexities in defining a learning disorder/disability, we defined learning challenge as any documented symptom affecting the child's learning. We found 14.2% of children ≥3 years of age with BPBI had information suggestive of a learning challenge noted in their HMR at a median age of 5 years. Given that this information is not routinely screened for in our BP clinic setting, where the focus is on BPBI-related physical abilities, our results may reflect an underestimation. These findings are similar to those of Buitenhuis et al (3), who found a 13% rate of central developmental disorder in their cohort, suggesting that the BPBI population is a high-risk group. Similarly, Bellew et al (12) found 44 preschool-aged children with BPBI had a higher rate of behavioural problems compared to typical peers (52% vs 3.3% to 11%). Despite the noted limitations in defining a learning challenge, these studies all describe inclusion of similar diagnoses (e.g., attention deficit disorder, behavioural challenges, cerebral palsy, developmental co-ordination disorder, etc.), supporting the finding that there is a higher rate of challenges with learning experienced by the BPBI population.

Of the collected demographic and treatment characteristics, only male sex was significantly associated with a learning challenge. We were unable to identify other literature commenting on differences by sex in children with BPBI and comorbidities. However, differences in physiological, biological, and social factors between boys and girls have been reported, and can be associated with higher levels of behavioural challenges in boys. These challenges may further have an impact on their learning (27).

While we did not look directly at the extent of nerve injury, children who spontaneously recovered by 6 months of age were excluded; therefore, our sample consisted of children who received more extensive BPBI treatment, and thus, were more likely to experience long-term functional changes. However, we found no significant association between interventions beyond traditional physiotherapy and learning challenges. These findings align with Buitenhuis et al (3), who found no association between the extent of nerve injury and central developmental disorder.

Lewis et al (14) identified a large gap in the literature for children with BPBI and a history of perinatal risk factors for central nervous system disorders, and the potential for long-term neurological co-morbidities. Within our study sample, children with a learning challenge had a higher rate of a documented birth complication compared to those without a learning challenge (61% vs. 40%). Although this was not statistically significant, it warrants further exploration given the potential for higher rates of birth hypoxia and asphyxia in infants with BPBI than the general population (8, 14), and their possible association with learning disorders (11).

From our sample, 10 of the 18 (56%) children with a documented learning challenge had a formal diagnosis (e.g., ADHD, ASD, or other learning disability), with an additional two children awaiting ASD assessment. Comments pertaining to challenges with attention, defiance, and impulsivity were noted for an additional six children with no formal diagnosis. Furthermore, 28% (5/18) noted comments of speech and/or language delays. This finding was similar to Chang et al (13), who found 30% of children with BPBI between 24 and 36 months of age had a language delay. Learning challenges in this study sample were first noted in the HMR at a median age of 5 years (IQR 3.5 to 10 years), though the age of detection could not be accurately identified. The age range of these findings is broad, but as Hayes et al (15) noted, challenges in learning can appear as early as 3 years of age, while Buitenhuis et al (3) commented on the potential for minor developmental problems to be diagnosed in an older child (> 8 years). In addition, children may present with physical, behavioural, or cognitive learning challenges, in and out of the classroom, long before or without ever meeting criteria for a formal learning disorder diagnosis. Thus, assessment and support should begin as soon as a learning challenge is identified (18, 19).

In line with Hayes et al (15), who noted challenges with defining and thus comparing learning challenges, it was difficult to identify a reference population for this study. However, the parameters of learning challenges described herein are most closely aligned with the definition of learning challenges used in the Canadian Health Survey on Children and Youth, 2023 (25). In contrast to the general population of Canadian children and youth aged 5 to 17 years old, we found a 10.9% greater rate of learning challenges in the BPBI study sample. While these results reflect our provincial center's experience with a small study sample, they support the findings of other studies, which demonstrated a higher prevalence of learning challenges experienced by children with BPBI (3, 12, 14).

A learning disorder is commonly diagnosed by a psychologist via a psychoeducational assessment; however, these processes are time-intensive, may have long waitlists (2+ years in Canada (20)), and can be costly, with some children never meeting criteria for a formal diagnosis. A screening program to facilitate timely identification of learning challenges and connection to local community and educational resources, with an overarching goal of optimizing a child's health and well-being, can begin with an HCP before formal testing is available/sought. The interdisciplinary BPBI team may be well positioned to consider implementation of informal screening for children at preschool (3 years) age and formal screening at 5 to 6 years of age, just before the child enters formal education, or at any time a family expresses concern. For example, resources such as the Ages and Stages Questionnaire (ASQ-3) (28) and the Parents Evaluation of Developmental Status Revised (PEDS-R) (29) are commonly used general developmental screening tools for children aged 1 month to five and a half years and birth to 8 years, respectively. Both instruments are parent-report measures that can be administered and scored quickly in the clinic setting.

This study was limited by a number of variables, including single-centre, missing data inherent to retrospective studies, and the inclusion of only children with long-term follow-up and thus with more severe BPBI. Although the best available reference population was used, variations between our sample and the reference population limited the comparison. No statistical comparisons of the sample and Canadian population were done as we could not control for a variety of variables, such as how or what learning challenges were reported in the HMR or by whom, how learning challenges were defined, or a child's age at time of reported learning challenge vs. age at identification.

This exploratory study suggests that children with persistent BPBI may have a higher prevalence of learning-related challenges compared to population estimates. However, differences in measurement methods, potential selection bias, and shared perinatal risk factors limit causal interpretation. These findings support consideration of structured developmental and learning surveillance within multidisciplinary BPBI care.
